# Early warning of infectious disease outbreaks on cattle-transport networks

**DOI:** 10.1371/journal.pone.0244999

**Published:** 2021-01-06

**Authors:** Frederik Schirdewahn, Hartmut H. K. Lentz, Vittoria Colizza, Andreas Koher, Philipp Hövel, Beatriz Vidondo

**Affiliations:** 1 Institut für Theoretische Physik, Technische Universität Berlin, Berlin, Germany; 2 Institute of Epidemiology, Friedrich-Loeffler-Institut, Greifswald - Insel Riems, Germany; 3 Sorbonne Universités, UPMC Univ Paris 06, INSERM, Institut Pierre Louis d’épidémiologie et de Santé Publique, Paris, France; 4 School of Mathematical Sciences, University College Cork, Cork, Ireland; 5 Veterinary Public Health Institute, University of Bern, Bern-Liebefeld, Switzerland; University of Lincoln, UNITED KINGDOM

## Abstract

Surveillance of infectious diseases in livestock is traditionally carried out at the farms, which are the typical units of epidemiological investigations and interventions. In Central and Western Europe, high-quality, long-term time series of animal transports have become available and this opens the possibility to new approaches like sentinel surveillance. By comparing a sentinel surveillance scheme based on markets to one based on farms, the primary aim of this paper is to identify the smallest set of sentinel holdings that would reliably and timely detect emergent disease outbreaks in Swiss cattle. Using a data-driven approach, we simulate the spread of infectious diseases according to the reported or available daily cattle transport data in Switzerland over a four year period. Investigating the efficiency of surveillance at either market or farm level, we find that the most efficient early warning surveillance system [the smallest set of sentinels that timely and reliably detect outbreaks (small outbreaks at detection, short detection delays)] would be based on the former, rather than the latter. We show that a detection probability of 86% can be achieved by monitoring all 137 markets in the network. Additional 250 farm sentinels—selected according to their risk—need to be placed under surveillance so that the probability of first hitting one of these farm sentinels is at least as high as the probability of first hitting a market. Combining all markets and 1000 farms with highest risk of infection, these two levels together will lead to a detection probability of 99%. We conclude that the design of animal surveillance systems greatly benefits from the use of the existing abundant and detailed animal transport data especially in the case of highly dynamic cattle transport networks. Sentinel surveillance approaches can be tailored to complement existing farm risk-based and syndromic surveillance approaches.

## Introduction

The transport of animals constitutes the backbone of the bovine industry. Animals are transported not only to slaughterhouses, but also between specialized rearing farms, to common pastures, to markets for trade and to exhibitions for evaluation and breading purposes. For dealers that facilitate the relocation of animals between farms, every trade is a source of revenue. For regions and countries that can be declared free of disease, free trade agreements exist to allow a relaxation of quarantine measures for the sake of a more agile economy and trade [1].

In Europe, animal transport networks have been shown to be highly heterogeneous [2–4]. In the case of the Swiss cattle, which will be the focal point of this paper, a small percentage of holdings is responsible for a large share of all animal transports [5, 6]. Furthermore, the incoming and outgoing number of contacts per holding are correlated, leading to amplifications in disease spread [7]. Besides, mass gathering events such as cattle markets are the prime risk factor for the spread of contagious diseases [6] and could potentially lead to super-spreading events (SSE, sensu, [8]) and thus, to explosive and large outbreaks. The role of markets has been recognized both in real outbreak studies (Foot and Mouth disease outbreak in Great Britain in 2001 [9]) and animal transport network studies [10–12]. The role of highly connected holdings in outbreak control has also been explored [13]. Surveillance and control programs at the national level are needed for the early detection and control of infectious diseases outbreaks. While earlier studies have pointed out the role of markets in control of outbreaks (e.g. [14, 15]), in this paper we focus on early warning.

In veterinary epidemiology, two different early warning approaches have been presented coming from different fields of research. On the one hand, the surveillance of syndromes (or syndromic surveillance) has recently been subject of a systematic review [16]. On the other hand, sentinel surveillance (informed by network analysis) is based on the analysis of animal transport data [6, 17, 18].

Syndromic surveillance is based on the (indirect) electronic monitoring and time series analysis of cases of syndromes (neurological, respiratory, digestive, also mortality and stillbirths data) within a time period. Several algorithms exist to estimate thresholds in the number of cases, above which an alarm is generated. The available data sets in Switzerland were evaluated by [19, 20] who found that data quality and reporting delays negatively affect the performance. Even though the sensitivity of the system can be maximized by fine tuning the data processing, moderate specificity (or non-negligible false alarm rates) remains [21]. The focus of this field of research seems to have shifted towards situation awareness [16].

Sentinel surveillance, on the other hand, is a direct approach based on the analysis of animal transport data (or social network analysis). It focuses on the selection of premises that would act as ‘guardians’ to detect threats or disease, hence, the name of ‘sentinel surveillance’. Two studies [17, 18] analyzed the overlap of transmission chains in the network to define clusters that reduce epidemic variability. A very small number (18 to 36) of sentinel holdings proved to detect 65% of outbreaks within 13 days in a network of about 100’000 nodes [18]. A further study [6] found that dynamic measures such as the outgoing contact chain resulted in ca. 83% median outbreak detection. In human epidemiology, two recent studies [22, 23] have evaluated different network-based surveillance strategies for early warning of outbreaks in temporal networks and found promising results in terms of the time difference (lead time) between the surveillance set and whole population in reaching 1% prevalence. Given the existence of long term time series of daily contacts for Swiss and European animal husbandry populations, sentinel surveillance provides an excellent approach that can be readily be applied.

Indeed, since 1999, when mandatory reporting of bovine transports was put into place in Europe and Switzerland [24], high quality time-stamped data at the animal level (from birth to death or slaughter) exists. Based on the chronology of the transports, potential chains of transmission can be reconstructed. This valuable information allows to shift the focus of surveillance programs from farms and geographical regions, to transmission chains for effective early warning and control [25].

Traditionally, farms are the epidemiological units where disease control is implemented as the animal owner can be held accountable. Once animal transport information becomes available, de-coupling surveillance from control becomes possible. This means that surveillance can be carried out at different types of premises or events, such as markets, and not only on the farms. This is especially advantageous for non-dairy farms that keep animals on semi-free range conditions on pastures, making sampling dangerous and time consuming [5]. A cross-sectional study that compared prevalence of sleeping sickness in villages and cattle markets in three districts in Uganda revealed that markets may be used as convenient sampling central points, especially for areas with emerging epidemics [26]. They found that in endemic areas, the prevalence at markets was higher than in the villages.

In the present study, we consider the case of cattle trade in Switzerland and propose a novel surveillance approach based on markets as proof of concept for an early warning system of emergent infectious diseases. By comparing a surveillance scheme based on markets to one based on farms, the aim of this paper is to identify the smallest set of sentinel holdings that would reliably and timely detect emergent disease outbreaks in Swiss cattle. In addition, we explore combinations of both markets and farms.

## Materials and methods

### Data sources

The animal movement database (in German *Tierverkehrsdatenbank* (TVD) or in French *Banque de données sur le traffic des animaux* (BDTA)) constitutes the nationwide registry for cattle and other animals in Switzerland. Further details on this database can be found in [5–7, 27]. We consider the Swiss in-country cattle transport data (excluding slaughter) over a period of four years starting at January 1 2012. The data set contains every transport of animals that occurred between two premises amounting to a total of 2,445,740 transport events and 49,497 reporting holdings. In addition to animal transport data, holding attributes are also available [28]. All holdings can be categorized by type as listed in Table 1. We focus on holdings labelled either ‘farm’ or ‘market’. The considered network is given as a directed time-dependent contact network with a holding of origin and a receiving holding. This time-dependent network defines a temporal, directed network *G* = (*V*, *E*), consisting of a set of nodes *V* and time-stamped edges *E*.

**Table 1:** Number of reporting holdings and their type in the Swiss cattle-transport network 2012-2015.

Type	Number of holdings
farm	42,515
market, auction and exposition	137
dealer	79
summering alp	6,721
clinic	7
unknown	38
total	49,497

### Outbreak model

We evaluate the surveillance schemes by simulating disease outbreaks starting from every holding on the first Monday of every month (48 months in the considered four years time series). We opt for a data-driven simple spread model to present and evaluate network dynamics in a transparent way. In this sense, our study aims for a proof of concept rather than to a detailed depiction of reality.

We consider a generic SIR model at farm level, where each holding can be either susceptible S, infected I or recovered R, assuming perfect detection and ignoring within-herd dynamics. For the purpose of our study, we focused on the number of sentinel holdings required to detect an outbreak as efficient (in terms of number of farms) and timely as possible. We evaluate our surveillance schemes using three criteria: detection probability, number of days until detection and number of infected premises at detection. While a fast spreading disease would reach a sentinel node in a shorter time, it would have also infected a higher number of other premises spreading through the network.

A susceptible holding becomes infected if it has contact to an infected one with probability *β*. After a fixed period *τ*, each infected holding becomes recovered from disease. The following transition scheme shows the disease progression of individual farms or holdings:

$$\begin{array}{c} S\overset{\;\beta \;}{\to }I\overset{\;\mu \;}{\to }R. \end{array}$$

At the start of the simulation, all holdings are susceptible. The infection starts at one single holding. *β* denotes the probability to get infected and *μ* recovery probability. We set *β* = 1, thus assuming that a contact always leads to an infection. The number of holdings (total population size) is assumed constant (i.e. no births and deaths) during the course of the outbreak. We consider the recovery probability per node and time step to be 1 (*μ* = 1) on the seventh day after infection, and zero otherwise. If outgoing connections from infected nodes happen within the infectious period of a holding, then the disease is transmitted to every susceptible receiving holding at each time-step. We opt to present results for *τ* = 7 so that the results are comparable to previous studies [17, 18]. The influence of the length of the infectious period on results was also assessed for a longer period (*τ* = 15) and our conclusions remained qualitatively unchanged. Of all possible outbreaks (starting from each of the 49,497 existing holdings times at 48 possible starting points) most outbreaks are smaller than 10 infected holdings and die out before detection, thus nullifying the need of early warning (and control). Including all outbreaks in our results would falsely decrease both the detection probability and the median outbreak size at detection. Thus, in order to calculate the detection probability, we consider only outbreaks of at least 10 infected holdings, which yields 43,842 outbreak events. We then calculate the detection probability as the proportion of all possible outbreaks (of at least 10 nodes) that infect at least one sentinel holding. Timeliness is measured as (1) the number of days elapsed since the start of the outbreak and (2) the number of holdings infected at time of detection. Note that for the longer infectious period considered, 15 days, the number of outbreaks larger than 10 holdings increases to 177,600, which considerably increased computational time and memory.

### Network metrics

We calculate the out-component *c*_out_(*v*_*i*_, *τ*, *t*_0_) of a node *v*_*i*_ as the set of nodes that can be reached from a primary infected node *v*_*i*_ ∈ *V* respecting the chronological order of the contacts for the finite infectious period *τ* equal to 7 days and for the total length of the time series starting at *t*_0_. This out-component corresponds to the final size of an outbreak originating from a specific node *v*_*i*_ [2]. Similarly, the in-component *c*_in_(*v*_*j*_, *τ*, *t*_0_) is the set of nodes from which a particular node *v*_*j*_ ∈ *V* can be infected respecting the chronological order of the contacts and for the finite infectious time period considered. The size of the in-component is a measure of the vulnerability (risk of getting infected) of a node [2]. For each holding, *t*_0_ is the time of the primary infection. Finally, the in- and out-degree of every farm is calculated as the number of incoming and outgoing transports of a farm with some other farm, for the four years time series. The sum of in- and out-degrees of a farm provides a measure of transport activity (and thus of acquiring and spreading the disease) of that particular farm.

### Surveillance schemes considered

Holdings are ranked and selected for surveillance according to (1) whether they are labelled as farms or markets, (2) largest sum of in- and out-degree, and (3) in addition, the largest vulnerability. In this way, we define a ‘market sentinel surveillance’ as a surveillance based on markets, which are ranked according to the sum of their in- and out-degree, and a surveillance based on farms or ‘farm sentinel surveillance’ based on farms only, ranked according to the sum of in- and out-degree and also according to the sum of in-degree, out-degree and vulnerability.

## Results

**Fig 1:**
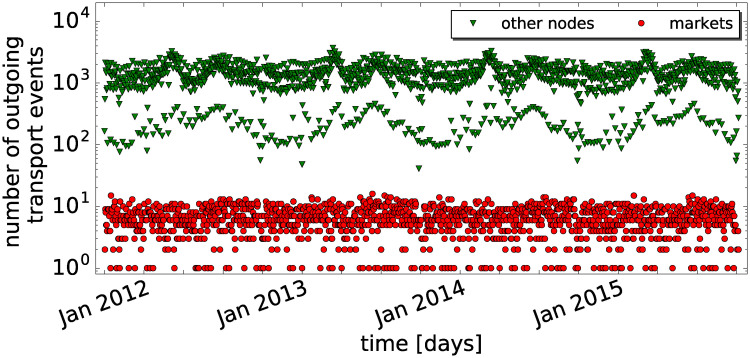
Daily outgoing transports in the Swiss cattle network. From 1.1.2012 to 31.12.2015, the sum of outgoing transport events that took place on each day for all markets (red dots) and all other holdings (green triangles) is plotted versus calendar time.

During the four years time series (1.1.2012 to 31.12.2015) the daily number of outgoing transport events for markets (red dots) and all other holdings (green triangles) remains stable (Fig 1). The seasonal peaks in early summer and autumn are due to transports to and from pastures and have been described in [6]. S1 Fig shows a long-term increasing trend for the number of transported animals with maximum values at the end of the series of 10^3^ for markets and 10^5^ for all other nodes. Both in terms of number of transports and number of animals transported, markets are responsible for a large share of the total traffic in spite of being in low numbers (only 137 markets during the observation period). Interestingly, traffic from the markets is fairly stable for any week and month of the time series.

**Fig 2:**
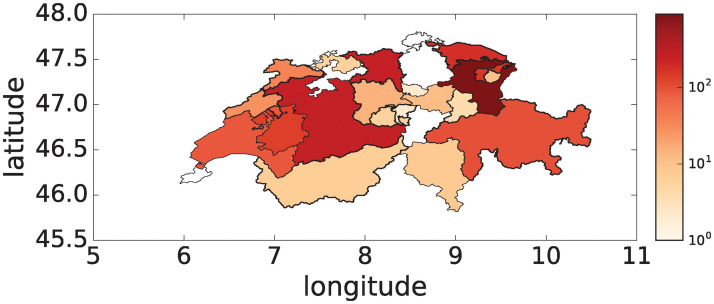
Map of Switzerland and outgoing transports per administrative region (canton). The color gradient indicates the sum of outgoing transports from the canton’s markets to other cantons (logarithmic scale, year 2012). The other years in the time series present a very similar pattern. The markets in the six cantons in white did not report outgoing transports to other regions (but were active otherwise). The data set excludes transport to slaughter. The map is created using publicly available information provided by opendata.swiss under BY licence [29].

Markets are active in each administrative region or canton both receiving and sending animals within and/or between cantons (Fig 2 and S2 and S3 Figs). Markets also deliver directly to slaughterhouses, usually to their own canton’s slaughterhouse. Since we excluded slaughter from the present analysis, six cantons did not show outgoing transports within their own canton.

**Fig 3:**
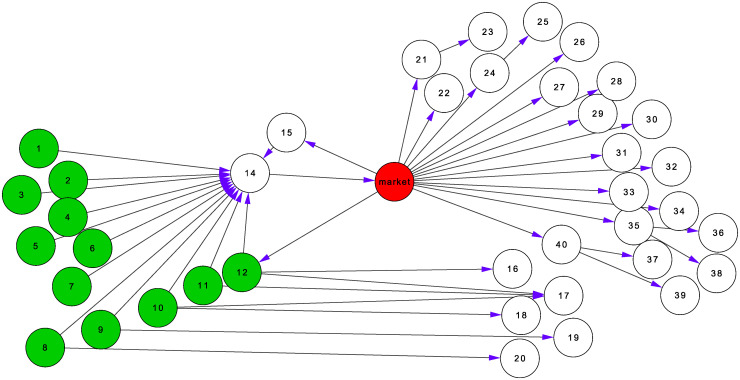
The role of markets in outbreak spread. Representation of one exemplary market (red node), which receives animals from 12 farms, that is, 12 different initial locations (green nodes), even though some transports are channelled via specialized farms to the markets (node 14). Five of these initial locations have additional trade contacts besides the depicted market. The outbreaks follow the direction of the arrows.

The role of markets in outbreak spread is schematically depicted in Fig 3, which shows an example of a market found in the data with its in- and out-component. If an infection occurs at any of the green nodes, the market (red) will be infected early and potentially cause an explosive outbreak. Placing the market under surveillance allows early detection of the infection and, provided control measures are implemented at the market, prevents the infection of downstream nodes via the market. Note that nodes may still be at risk via other transmission pathways. See, for instance, the five white nodes on the lower right that are reached directly from the initially infected nodes.

**Fig 4:**
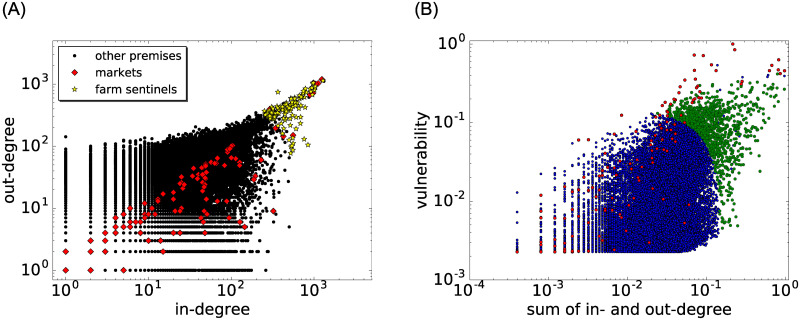
Scatter plots of degrees and vulnerability. Left panel: Positive trend of the out-degree versus the in-degree of each holding. Markets (red diamonds), farms with largest in- and out-degree (yellow stars), all other holdings (black dots). Right panel: Positive trend of the vulnerability with the sum of the degrees. Markets (red circles), farms with the largest vulnerability and largest sum of degrees (green dots), all other holdings (blue). Both vulnerability and sum of degrees are normalized by their maximum values.

The in- and out-degrees (Fig 4 (left)) of markets (red diamonds) and farms (with largest in- and out-degree marked by yellow stars) show a positive trend. Similarly, the vulnerability increases with the sum of in- and out-degree (Fig 4 (right)). This increase is steeper for markets (red dots). For the farm surveillance, farms were ranked according to the sum of in- and out-degree and also according to the sum of in-degree, out-degree and vulnerability, and selected starting from the maximum of either of these two criteria. Note that there are some blue nodes among the green group. They correspond to non-farm premises such as clinics, where tests are already performed, or summering alps that are only inhabited during the summer months and thus, are less suitable for surveillance [6].

**Fig 5:**
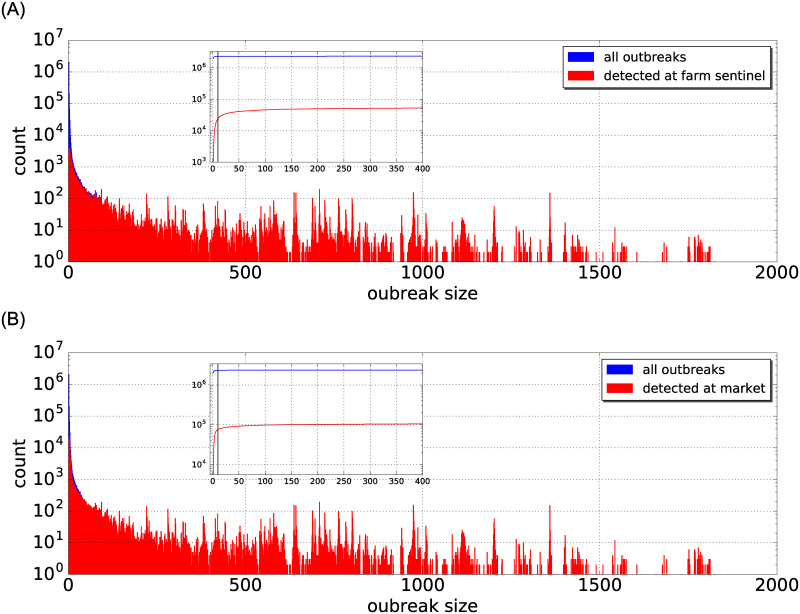
Distribution of outbreak size. Histograms of sizes of all outbreaks (blue) and of outbreaks detected by farm sentinel surveillance (top panel, red) and market surveillance (bottom panel, red). The insets show the cumulative distributions of outbreak size for all outbreaks (blue curves), farm sentinel surveillance (top panel, red curves), and market surveillance (bottom panel, red curve). The vertical lines in the insets mark the outbreak size equal to 10 infected holdings as a reference.

The distributions of outbreak sizes for both farm surveillance and market surveillance are skewed towards low numbers as shown in the top and bottom panels of Fig 5, respectively. In this figure, 137 farm sentinels are selected according to the largest sum of in- and out-degree. Large outbreaks can be detected by either farm sentinels or market sentinels. The insets show the cumulative distribution of outbreak sizes. The vertical line marks an outbreak size of 10 holdings as a reference. It indicates that the vast majority of possible outbreaks (2,332,062 or equivalently 98.2%) is very small (10 holdings or less) and will not affect large parts of the network. In the following, we focus on outbreaks that include at least 10 holdings.

**Fig 6:**
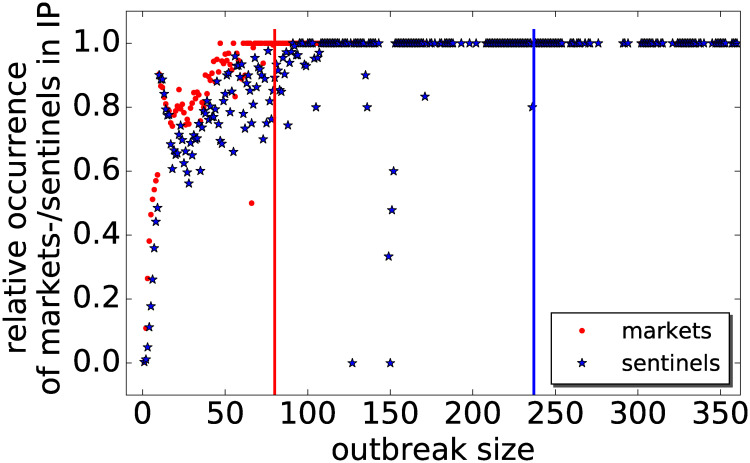
Probability of finding a market (red dots) and farm sentinel (blue stars) versus outbreak size. The vertical lines indicate the outbreak size above which 100% detection is achieved (red line for markets at an outbreak size of 80 premises, blue line for farm sentinels at an outbreak size of 237 premises).

The probability of finding a node, which has been selected for monitoring, becomes larger with increasing outbreak size (Fig 6). The larger the outbreak, the more likely it is that it hits a node under surveillance. This probability increases more rapidly for markets (red dots) than for farm sentinels (blue stars). It reaches 100% for an outbreak size of 80 (red vertical line), whereas for farm sentinels the 100% mark is reached above an outbreak size of 240 (blue vertical line). In other words, any outbreak larger than 80 contains a market. This explains the better performance of market sentinels compared to farm sentinels.

The better performance of markets can be further explored by quantifying the time until detection. We find that a higher number of outbreaks is detected earlier and at smaller outbreak sizes for the surveillance at 137 markets than for the farm surveillance with the same number of sentinels (cf. S4 Fig).

**Fig 7:**
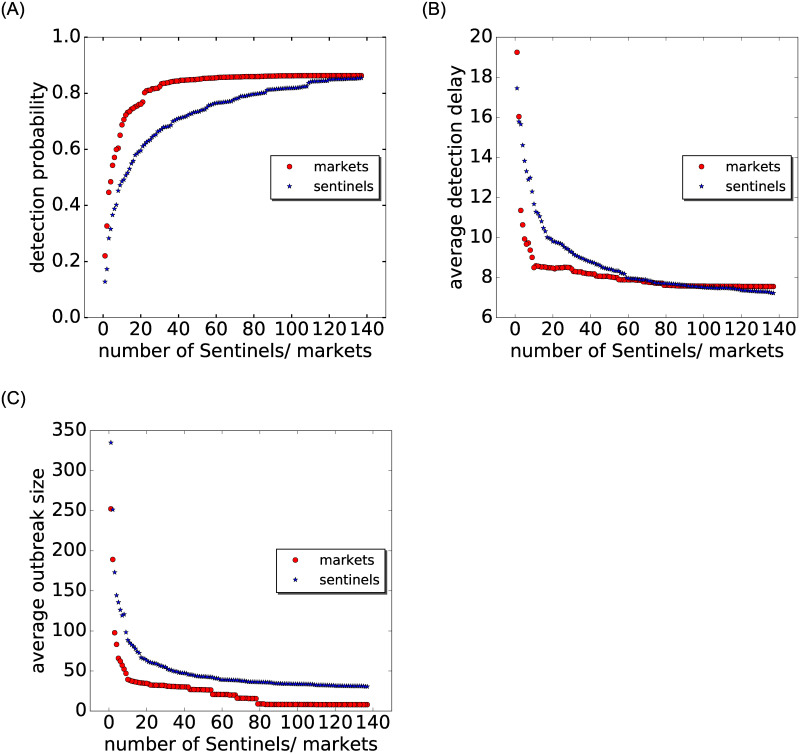
Detection probability, detection delay and outbreak size at detection. From left to right: Detection probability, average detection delay (days) and average number of infected nodes (until detection) for an increasing number of sentinel farms and markets; market surveillance (red circles) and farm sentinel surveillance (blue stars). Both markets and farm sentinels are sorted by highest sum of in- and out-degree.

To corroborate the performance of both surveillance schemes, Fig 7 depicts three measures in dependence on the number of sentinel nodes, which increases from 1 to 137: detection probability (left panel), time until detection (center panel) and outbreak size at detection (right panel). We again observe that the market surveillance performs better (higher detection probabilities, lower detection delays and lower outbreak sizes at detection). This holds in particular for smaller numbers of nodes under surveillance. For market surveillance, the detection probability reaches a high plateau at around 80% with as few as 20 markets under surveillance. The average detection delay plateaus at 7 days for both schemes above 60 sentinel nodes. The outbreak sizes at detection are consistently around 25 infected nodes lower for market surveillance than for farm surveillance. The average outbreak size becomes minimal (8 vs. 31 infected nodes for market and farm sentinel surveillance, respectively) with 80 markets or more under surveillance.

**Fig 8:**
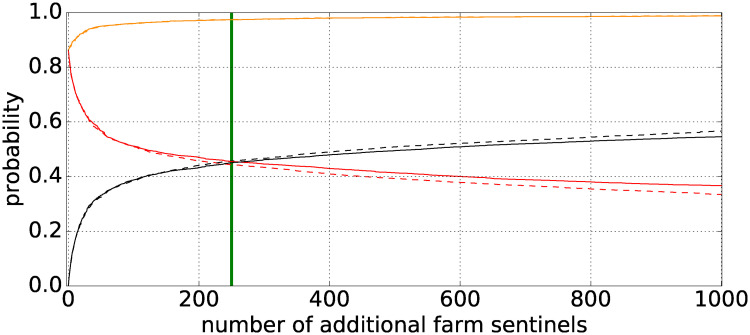
Probability of first hitting a market or a farm sentinel. The red and black curves show the probability of outbreaks to first hit a market or a farm sentinel, respectively. The green vertical line marks the crossover, when a farm sentinel is detected first. Schemes are based on the highest sum of in- and out-degree (solid curves) and in addition, the largest vulnerability (dashed curves). Detection probability (orange curve) starting with 137 markets and adding one farm sentinel at a time.

Up to this point, we have considered the sum of in- and out-degree as a measure to identify adequate farm sentinels for surveillance. An alternative approach is to account for nodes with a high vulnerability. These are nodes that are infected from many sources and serve as promising points of monitoring. Thus, we explore the maximum detection probability that can be achieved by starting with 137 sentinel markets and adding—one at a time—additional sentinel farms up to 1,000 (cf. Fig 8). Placing additional sentinel farms under surveillance is done according to (i) the largest sum of in- and out-degree (solid curves) and (ii) the largest vulnerability as an additional criterion (dashed curves). The total detection probability (orange curve) increases progressively to a maximum of 99%. This means that over the course of the four years observation period, almost every possible outbreak larger than 10 farms, could have been detected with 137 markets and 1,000 additional farm sentinels. Besides, the average detection delay is reduced from 7 days (cf. Fig 7) to 4.2 days and the average outbreak size at detection is reduced from 8 (cf. Fig 7) to 3.3 holdings. An additional effect of adding farm sentinels is that the outbreaks can be detected at a farm (instead of at a market). As the number of additional farm sentinel increases, the probability of first hitting a farm (black curves) increases as well. The probability of first hitting a farm equals the probability of first hitting a market at 250 additional sentinel farms (green vertical line in Fig 8).

## Discussion

Our results show that surveillance placed on markets leads to an earlier detection of outbreaks compared with surveillance placed on farms—and this even with a smaller number of nodes. Moreover, the detection probability is much higher for market based surveillance, and the outbreaks are detected earlier on average, both in terms of detection delay (8 days) and outbreak size at detection (ca. 20 infected holdings) (cf. Fig 7). Since markets typically occur early in infection paths in our simulations (cf. Fig 6), they are suitable points for an effective outbreak detection. In order to assure the overall coverage of markets, we have shown that markets cover the trade network in terms of their temporal (cf. Fig 1) and spatial presence (cf. Fig 2, S3 Fig).

The idea of using animal transport data to inform risk-surveillance programs (although not necessarily for early warning) has been discussed in Frössling et al. [30]. The authors propose to calculate the probability of disease of a particular destination herd, based on (i) animal transports of specific (relevant) time periods, and (ii) the prevalence of disease on different levels (typically within herd and between herds). They calculate the *probability of disease ratio*, as a ratio that measures the relative increase in probability of disease. However, these calculations assume a non-negligible prevalence and are therefore not adequate for early warning.

In this paper we have used node attributes from the data to label markets as such. It should be noted that the term ‘market’ as it is used here encompasses a variety of animal and animal holder gatherings for various purposes (such as expositions or auctions). Even though some European countries have abolished trade or slaughter-markets as such (e.g. Germany or Sweden), other animal gatherings like expositions or fairs do exist in European countries. Within the trade network all the mentioned events act as markets from a topological perspective, that is, they have a similar position within the network.

Our simple epidemic model allows us to evaluate the network properties in a transparent way. Other factors like additional time delays (in reporting of the transport data, or reporting of suspected cases, or the laboratory confirmation of the suspected cases), detection compliance, within-herd dynamics, varying herd sizes and test efficiency have not been taken into account. A disease-specific model with within-herd dynamics and varying scenarios could address these aspects in the future.

The present work is a proof of concept and of rather theoretical nature. From a more practical perspective, the role of markets as sentinels has been discussed in [26] for an Ugandan trade network. The authors found that infected animals accumulate at markets due to the fact that farmers try to repeatedly sell low-performing animals. This higher prevalence at the markets can be advantageous for early warning. Similar to risk surveillance, sampling at such markets means sampling at places with a higher probability to find infected animals.

In a practical context, the selection of sentinels depends on the public health goal, the network topology, and the properties of the considered disease [22]. A further criterion is data availability, as it allows for the evaluation of different schemes and also determines whether a scheme is realistic or not. Our results highlight the importance of the holding categories data being available, in addition to the transport data. The public health goal chosen in the present study is early warning and we show that reliable outbreak detection at farm level (99% detection probability) is possible at very low farm prevalence. This is well below the limits of the free trade agreement of 0.1% (necessary to substantiate freedom of disease, [1]) (cf. Results: average outbreak size at detection 3.3 infected holdings out of 49,497, i.e. 0.008% prevalence at detection).

## Conclusion

We conclude that especially in the case of highly dynamic cattle transport networks the design of animal surveillance systems would greatly benefit from the use of the abundant and detailed animal transport data available. Sentinel surveillance approaches can be tailored to complement other farm risk-based and syndromic surveillance approaches. Specifically, Switzerland’s early warning system would profit from combining surveillance at farms with that at markets. Future studies should address the cost-effectiveness and practicability of surveillance based at markets for specific diseases, both emergent or production diseases.

## Supporting information

S1 Fig Daily outgoing number of animals in the Swiss cattle network. From 1.1.2012 to 31.12.2015, the total number of outgoing animals that took place in each day for all markets (red dots) and all other holdings (green triangles).
(EPS)

S2 Fig Map of Switzerland and cattle transports per administrative region (canton). Transports within canton (size of orange circles) and transports to other cantons (size of black arrows) from each canton’s markets (logarithmic scale—year 2012). The data set excludes slaughter. All other years follow a very similar pattern. Canton St. Gall had the largest sum and is plotted in separately in S3 Fig. The map is created using publicly available information provided by opendata.swiss under BY licence [29].
(EPS)

S3 Fig Map of Switzerland and market cattle transports, canton St. Gall. Transports within each canton (size of orange circles) and transports to other cantons (size of black arrows) from the markets of canton St Gall (logarithmic scale—only year 2012). All other years present a similar pattern. The data set excludes transport to slaughter. The map is created using publicly available information provided by opendata.swiss under BY licence [29].
(EPS)

S4 Fig Detection performance of market vs. farm sentinels. Heat maps of the number of detected outbreaks in relation to outbreak size at detection and detection time in days for both surveillance schemes: 137 farm sentinels (left panel) and 137 markets (right panel).
(EPS)
